# Reaction Behavior of Ultrafine Ferric Oxide Powder with Hydrogen–Carbon Monoxide Gas Mixture

**DOI:** 10.3390/ma18215002

**Published:** 2025-11-01

**Authors:** Xudong Mao

**Affiliations:** 1 School of Resources & Environment, Nanchang University, Nanchang 330031, China; maoxudong_ustb@163.com; 2 Jiangxi General Institute of Testing and Certification, Nanchang 330052, China; 3 State Key Laboratory of Advanced Metallurgy, University of Science and Technology Beijing, Beijing 100083, China

**Keywords:** iron oxide, reduction reaction, carbon monoxide, hydrogen, kinetics mechanism

## Abstract

This study aims to enhance fundamental research on the reaction behavior between ferric oxide and H_2_–CO gas mixtures and to provide theoretical support for optimizing the injection of hydrogen-containing materials in the ironmaking process. In this study, the ultrafine ferric oxide powder was isothermally reduced with H_2_–CO gas mixture at 1023 K–1373 K. The results indicated that when H_2_ content is less than 30% at 1023 K, the ferric oxide sample reduced by the H_2_–CO gas mixture exhibits a pronounced carbon deposition phenomenon during the reduction stage. The gas reactant composition had a relatively large influence on the reaction rate at the third stage of the reduction reaction (FeO → Fe). Assuming the single-step nucleation assumption theory together with kinetic experimental data, the relationship between the average reaction rate and the gas composition of the H_2_–CO gas mixture was established for the FeO reduction stage. In addition, the apparent activation energy of the reduction reaction was generally in the range of 20–45 kJ/mol, indicating that the possible rate-controlling step was combined gas diffusion and interfacial gas–solid chemical reaction.

## 1. Introduction

For the current steel industry, the energy consumed in ironmaking is mainly carbonaceous energy and accompanied by a large amount of CO_2_, and the excessive emission of CO_2_ will have a serious impact on the environment [1,2,3,4,5,6]. In order to reduce the emission of CO_2_ in the ironmaking process, it may be a better way to use hydrogen-containing materials to replace part of the carbonaceous materials at present [7,8], in which the reduction behavior of iron ore under H_2_–CO atmosphere will be involved. Therefore, it is necessary to systematically study the reaction behavior of iron oxides with H_2_–CO gas mixture.

According to fundamental principles of reduction kinetics, in metallurgical processes, the reduction behavior of metal oxides (including iron oxides) is generally governed by a complex interplay among multiple parameters, primarily including the composition and flow rate of the gas reactant, the inherent physical properties of the iron oxide, and the reaction temperature. Du et al. [9] investigated the reduction behavior of hematite powder to magnetite by H_2_ at low temperatures, and the experimental results demonstrated that below 673 K, the reduction reaction’s possible rate-controlling step was controlled by the interfacial chemical reaction. However, when the temperature exceeded 723 K, the possible controlling mechanism shifted to gas diffusion. Yi et al. [10] monitored the volumetric changes in iron ore pellets during reduction by H_2_–CO gas mixture at 1073 K to 1273 K, and the results revealed that the ore pellet expansion was enhanced with increasing temperature and CO concentration, reaching its maximum rate when the reduction degree was 20–40% due to the formation of the wustite phase and pellet expansion. Jeongseog et al. [11] examined the structure of the metallic iron product formed during the reduction of hematite by an H_2_–CO gas mixture. Compared to the structure produced by pure H_2_ reduction at 1073 K, the metallic iron generated using CO exhibited a more friable structure and less inter-particle sintering, which can be attributed to the carbon deposition during CO reaction and the difference in diffusion behavior and reduction behavior between H_2_ and CO.

It is evident from the current published research that studies on the reduction of iron oxides by H_2_–CO gas mixture predominantly focus on conventionally scaled materials. Rajakumar et al. [12] systematically investigated the reduction of wustite particles with particle sizes of 100 μm, 200 μm, 300 μm, and 400 μm by H_2_–CO gas mixtures at 1173 K–1373 K, and the results revealed that under H_2_ reduction conditions, abundant metallic iron nuclei formed on the wustite surface, whereas fewer nuclei generated under CO reduction, but the growth rate of iron nuclei was faster than that of H_2_. EI-Geassy et al. [13] examined the reduction behavior of wustite pellets (150–250 μm) using H_2_–CO gas mixture at 1173 K–1373 K, and demonstrated that introducing 5% CO into pure H_2_ gas reactant would rapidly reduce the rate of reduction reaction. However, when the CO content in the H_2_–CO gas mixture exceeded 25%, the difference in the reduction rate between the H_2_–CO and H_2_–Ar atmospheres diminished. The main influence mechanism was that once CO was adsorbed on the active site of the wustite, it occupied the adsorption site of H_2_ at low CO content, which hindered the reaction. When CO content was greater than 25%, the surface active sites were basically covered by CO, so the effect of further increasing CO on the active sites was limited, thus reducing the reaction rate difference. Fruehan et al. [14] studied the reduction kinetics of iron oxide with different crystal sizes under H_2_ atmosphere. The results indicated that for wustite with a grain size of approximately 1 μm, the reduction by H_2_ showed negligible deceleration until a reduction degree of 95% was reached. In contrast, for samples with a grain size around 2 μm, the reduction rate decreased significantly even before the possible rate-controlling step shifted to gas diffusion. This study further proposed two kinetic mechanisms, provisionally designated as the gas-reduction kinetic equation and the solid-state diffusion kinetic equation, and identified the criteria for switching between these mechanisms based on the grain size of wustite and the thickness of the generated iron layer. For wustite grains around 1 μm in size, the gas-reduction kinetic equation remained applicable up to nearly complete reduction (>99% reduction degree); whereas for grains of 2 μm and larger, it was necessary to switch to the solid-state diffusion equation when the iron layer thickness was about 1 μm and the reduction degree reached 60–70%. Thus, it is noteworthy that relatively significant research gaps remain concerning the reaction behavior of ultrafine iron oxide powder (<1 μm) with H_2_–CO gas mixture, and addressing this knowledge gap is crucial for developing low-carbon and efficient ironmaking technologies. Based on this situation, the present work conducts systematic experiments on the reduction of ultrafine ferric oxide powder (with the particle size range <1 μm) by H_2_–CO gas mixture, and further improves the basic research on the reaction behavior of iron oxide and H_2_–CO gas mixture. The purpose is to clarify the reaction kinetics and apparent activation energy of the reaction under low particle size, and to provide theoretical support for metallurgical process optimization.

## 2. Materials and Methods

### 2.1. Experimental Materials and Setup

**Figure 1:**
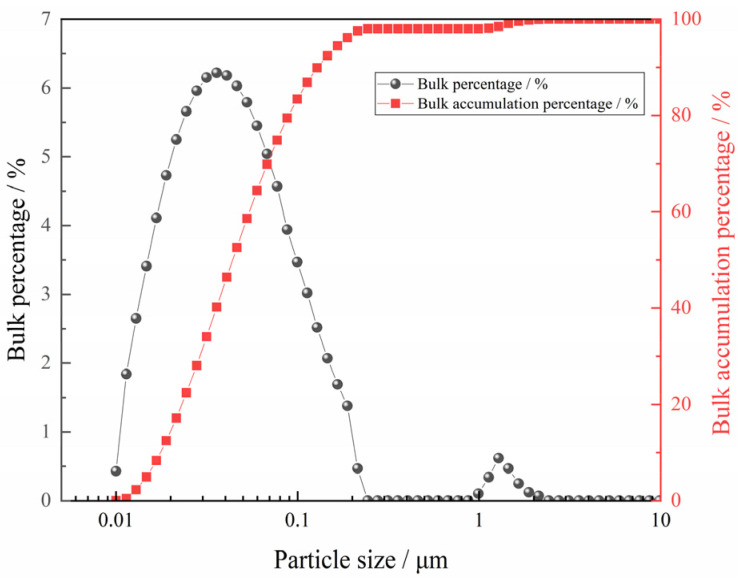
The particle size distribution of the sample.

The experimental sample used in this study was reagent-grade Fe_2_O_3_ powder (99.9%). Before the experiment, the sample was placed in a tube furnace at 773 K for 1 h to remove any possible water, and then placed in a vacuum drying oven at 473 K to prevent the sample from absorbing water. The particle size distribution of the ferric oxide sample used in the experiment was measured by the laser particle size analyzer (CLF-2, Malvern, Great Malvern, UK). It can be found that the particle size is basically less than 0.2 μm, and the detailed result is shown in Figure 1.

**Figure 2:**
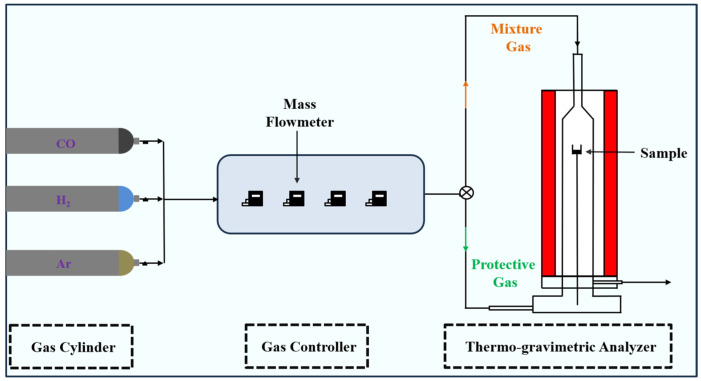
Experimental setup.

The schematic diagram of the experimental setup is given in Figure 2. In this experiment, a thermogravimetric analyzer (TGA, HCT-4, Henven, Beijing, China) was used to study the reaction behavior of the ferric oxide sample with H_2_–CO gas mixture. The 100 mg sample was placed in an alumina crucible (20 mm height, 16 mm inner diameter) to form a thin layer to prevent the material layer from being too thick and affecting the experimental results. The alumina crucible was cleaned by an ultrasonic cleaner and then desiccated by a vacuum desiccator before use. The sample was heated to the experimental preset temperature under the protection of Ar atmosphere (Praxair, Beijing, China, LR ≥ 99.999%), and then introduced to H_2_–CO (Praxair, Beijing, China, LR ≥ 99.99%) gas mixture for isothermal reaction experiment, which the gas flow rate of each gas was accurately controlled using the mass flowmeter (Alicat Scientific, Tucson, AZ, USA).

According to previous studies [15,16,17], it can be found that under the same experimental equipment conditions, when the gas flow rate is 40 mL/min, the effect of gas external diffusion on the reaction can be significantly reduced. Therefore, in this study, the gas flow rate of 40 mL/min was also employed, and temperature varied in the range of 1023 K–1373 K. Table 1 shows the experimental conditions employed in the present investigation, which systematically covers different temperatures (1023 K, 1173 K, 1273 K, 1373 K) and various H_2_/CO flow rate ratios (with the total flow rate maintained at 40 mL/min, including pure CO, pure H_2_, and intermediate H_2_/CO proportions). The physical phase composition of the reaction products was characterized using a scanning electron microscope (SEM, MLA250, FEI, Hillsboro, OR, USA) and X-ray diffraction (XRD, SmartLab, Rigaku, Tokyo, Japan), respectively.

During the reaction of the sample with H_2_–CO gas mixture, the mass loss of the sample is caused by oxide reduction, either by chemical action of H_2_ or CO. Conversely, the mass increase can occur under specific conditions due to carbon deposition or cementite formation. This is primarily associated with the Boudouard reaction, which leads to graphite formation and the carburization of metallic iron, resulting in cementite formation. The prevalence of these reactions is highly dependent on the gas composition and temperature. In order to better study the reaction behavior of the sample, the apparent reaction extent, *X*, of ferric oxide sample was defined as the ratio of the mass change in the sample at time t to the theoretical oxygen loss mass of the reaction, which the theoretical oxygen loss mass was calculated based on the complete reduction stoichiometry of Fe_2_O_3_ to Fe (Fe_2_O_3_ → 2Fe + 1.5O_2_), corresponding to 30% mass loss of the initial sample.

(1) $$X=\frac{m_{i}-m_{t}}{m_{o}}$$

where *m_o_, m_i_*, and *m_t_* are the theoretical oxygen loss mass, the mass of the initial sample, and the sample at time t, respectively. The average reaction rate, *v*, can be obtained by the following formula:

(2) $$v=\frac{∆X}{∆t}$$

### 2.2. Thermodynamics Analysis

**Figure 3:**
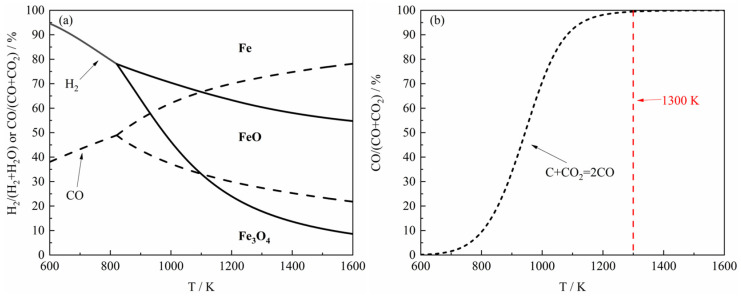
The thermodynamic phase diagram at 1 atm: (**a**) the Fe–O–H (or C) system equilibrium; (**b**) the Boudouard reaction system equilibrium.

The whole process of the reaction of the ferric oxide sample with H_2_–CO gas mixture can be roughly divided into the reduction part and carbon deposition part (which may not exist under certain conditions) [18,19], and the reduction of ferric oxide to metal iron is also divided into several reduction steps [20,21,22,23]. The thermodynamic phase diagram for the Fe–O–H and Fe–O–C system equilibrium, calculated with FactSage 8.0 calculation software (Thermfact/CRCT, Montreal, Canada, and GTT-Technologies, Aachen, Germany), is shown in Figure 3, and the ordinate of the diagram represents the mole fraction percentage of H_2_/(H_2_ + H_2_O) (for the Fe–O–H system) or CO/(CO + CO_2_) (for the Fe–O–C system). From Figure 3a, it can be observed that in the Fe–O–H system, the reduction capacity of H_2_ gradually increases when the reaction temperature increases, as only a lower mole fraction of H_2_ is required to achieve the same reduction degree at higher temperatures. In contrast, in the Fe–O–C system, the reduction capacity of CO shows a decreasing trend with the rise of reaction temperature, since a higher mole fraction of CO is needed to maintain the reduction degree. In addition, as shown in Figure 3b, when the temperature increases to 1300 K, carbon deposition requires an extremely high CO mole fraction (close to 100%). This thermodynamic constraint indicates that the carbon deposition is strongly suppressed even in the presence of a small proportion of CO_2_ under common CO experimental conditions.

## 3. Results and Discussion

**Figure 4:**
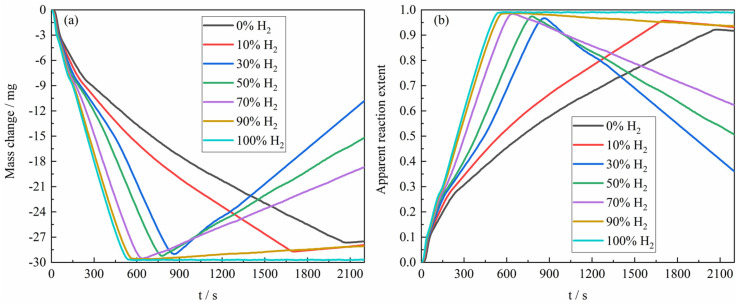
The reduction curves for the sample with H_2_–CO gas mixture at 1023 K: (**a**) sample mass change; (**b**) apparent reaction extent.

Figure 4a presents the mass change curves during the reduction of the ferric oxide sample by H_2_–CO gas mixture with varying composition ratios at 1023 K. When the gas reactant only contained pure H_2_, the sample exhibited the greatest mass loss, approaching the theoretical oxygen loss of 30 mg for the complete reduction of 100 mg Fe_2_O_3_ to Fe. As the CO content in the H_2_–CO gas mixture increased, the mass loss of reaction gradually decreased, reaching only −27.6 mg under pure CO. The reduction process consisted of three distinct stages (Fe_2_O_3_ → Fe_3_O_4_, Fe_3_O_4_ → FeO, and FeO → Fe), which correspond, respectively, to mass changes in approximately 0 to −3 mg (Fe_2_O_3_ → Fe_3_O_4_) and −3 to −9 mg (Fe_3_O_4_ → FeO), and the average reaction rate at different stages of the reaction would change accordingly. In the later stages of reaction, carbon deposition occurred via the Boudouard reaction (2CO → C + CO_2_), which corresponded to the mass increase of the reaction sample in the figure. Furthermore, the experimental results revealed that the rate of the carbon deposition reaction would tend to increase first and then decrease with the increase of the proportion of CO in the H_2_–CO gas mixture, which can be attributed to the dual role of H_2_. The main reason is that the presence of H_2_ promotes the carbon deposition [19] by facilitating the formation of an active intermediate during the Boudouard reaction. However, when the H_2_ content in the H_2_–CO gas mixture is relatively high, the CO content, as the main reactant of carbon deposition, is significantly reduced, resulting in a decrease in the carbon deposition rate. In addition, the metallic Fe formed under a relatively high H_2_ atmosphere is prone to sintering densification, which reduces the effective surface area for carbon deposition and further reduces the rate and degree of carbon deposition. In addition, the conversion of sample mass change to apparent reaction extent was carried out according to Formula (1), and the results of apparent reaction extent for the reaction of H_2_–CO gas mixture of different composition ratios with ferric oxide sample at 1023 K were presented in Figure 4b.

**Figure 5:**
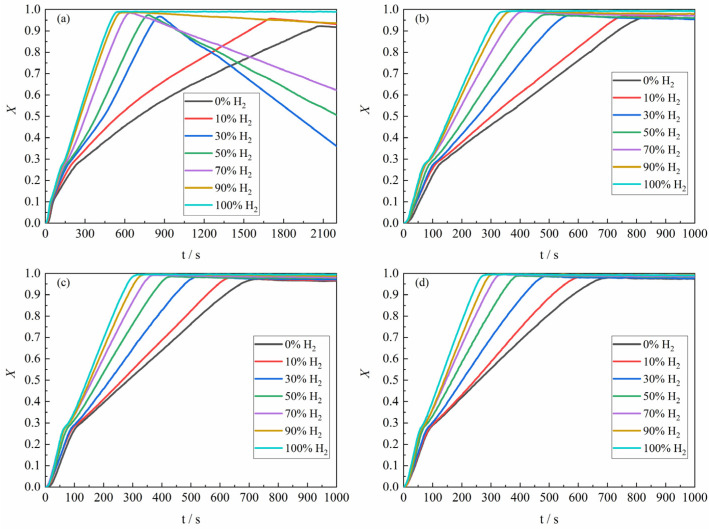
The apparent reaction extent curves for the sample with H_2_–CO gas mixture: (**a**) 1023 K; (**b**) 1173 K; (**c**) 1273 K; (**d**) 1373 K.

As shown in Figure 5, the overall experimental results for the apparent reaction extent of H_2_–CO gas mixture of different composition ratios with ferric oxide sample at 1023 K–1373 K were demonstrated. It can be found from Figure 5b that the higher the proportion of H_2_ in the H_2_–CO gas mixture, the closer the mass change in the ferric oxide sample was to the theoretical maximum mass loss, which was consistent with the reaction condition at 1023 K. Moreover, when the gas reactant only contained pure CO, the maximum apparent reaction extent was higher than the apparent reaction extent of pure CO reducing ferric oxide sample at 1023 K. The apparent reaction extent curves of the ferric oxide sample with H_2_–CO gas mixture at 1273 K and 1373 K are shown in Figure 5c,d.

In summary, the reaction of the ferric oxide sample by the H_2_–CO gas mixture during the reduction stage basically exhibited a consistent trend in apparent reaction extent curves. The time required to reach maximum mass loss decreased with increasing H_2_ content, where the largest decrease was in the period of the increase of the H_2_ content from 0% to 30%. At 1373 K, the reaction of the ferric oxide sample was relatively complete, and the H_2_–CO gas mixture basically could reduce the ferric oxide sample to metallic iron, though the time required was similar to that at 1273 K. Figure 5d indicated that carbon deposition was largely suppressed at 1373 K, consistent with the thermodynamic trend shown in Figure 3. Additionally, the maximum apparent volume of the product from CO reduction was approximately four times greater than that from H_2_ reduction, as determined by measuring the apparent volume of the product sample in the crucible after reaction, in agreement with the results reported by Nasr et al. [10,24,25], further confirming more severe sintering under H_2_ atmosphere.

**Figure 6:**
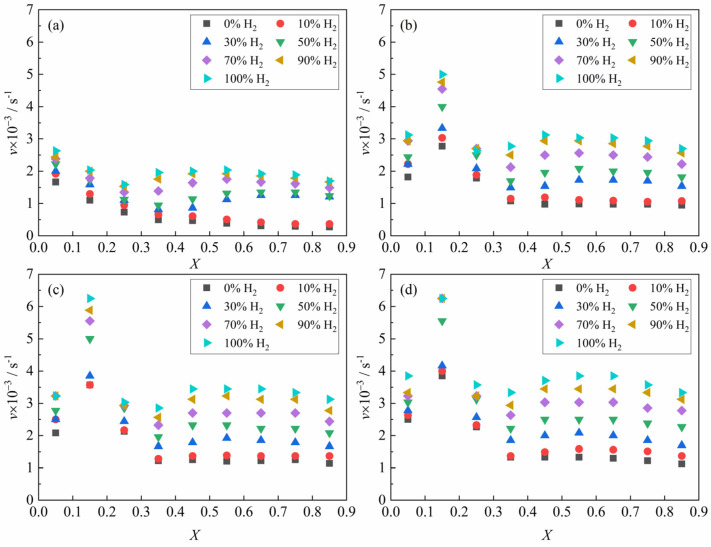
The curves for average reaction rate with apparent reaction extent under different conditions: (**a**) 1023 K; (**b**) 1173 K; (**c**) 1273 K; (**d**) 1373 K.

Figure 6 presents the relationship between the average reaction rate of the ferric oxide sample with the H_2_–CO gas mixture and the apparent reaction extent under different conditions. At 1023 K, the average reaction rate decreased continuously within the apparent reaction extent range of 0–0.3 (Fe_2_O_3_ → Fe_3_O_4_ → FeO). At temperatures ≥1173 K, the average reaction rate first increased and then decreased. When the apparent reaction extent exceeded 0.3, the reaction entered the third reduction stage (FeO → Fe), which has been identified in Figure 5 and previous studies [19,20,26,27] as the rate-controlling stage of the overall process. Based on the apparent reaction extent curves and average reaction rate of the third reduction stage (FeO → Fe), this process can be divided into three periods [28]: the incubation period, the acceleration period, and the exhaustion period. Referring to the systematic analysis of the reduction morphology of FeO by John et al. [29,30] and combining the results of the present experiments, it was found that in the initial stage of FeO reduction, pits formed on the FeO surface before extensive aggregation of metallic iron clusters. The main reason may be caused by lattice vacancy formation due to oxygen loss. During the incubation period, the gas reactant reacted with the oxygen atom in the FeO at the reaction interface and brought it out, while the oxygen atom in the FeO inside the reaction interface needed to diffuse to the reaction interface first and then reacted with the gas reactant. As the reaction progressed, iron atoms initially covering the reaction interface in localized regions of the sample agglomerated and grew, thereby exposing fresh FeO surfaces. And the gas reactant could directly contact the surface of the fresh FeO and react, causing the local reaction rate to be accelerated. When the whole sample showed this phenomenon, the macroscopic performance was that the reaction rate increased significantly, which means that the incubation period was over and the acceleration period had entered. During the acceleration period, the reaction interface continued to expand into the interior of the sample, causing the reaction interface to continuously increase. Due to the large consumption of FeO in the sample, the reaction finally entered the exhaustion period. In addition, according to Figure 5 and other research results [31], part of the iron oxide was tightly surrounded by dense metal iron and was difficult to reduce under certain reaction conditions in the exhaustion period.

During the reaction, it was assumed that the reduction of the sample with the gas reactant to produce a single iron atom was equivalent to the formation of a single-step nucleus, and then the overall nucleation rate was the reaction rate of the sample. According to the Jacobs single-step nucleation assumption theory [32,33], the rate of nucleation, $v_{t}$, can be derived using the following formula [19]:

(3) $$v_{t}=\frac{dN}{dt}=K_{N}(N_{0}-N)$$

where $K_{N}$ and $N_{0}$ represent the rate constant of nucleation and the potential nucleation sites assuming equivalent probability of nucleation, and N is the number of active growth nuclei at time *t*, respectively. Integration of Formula (3) using variable separation, the result can be obtained:

(4) $$N=N_{0}(1-e^{-K_{N}t})$$

Substituting Formula (4) back into Formula (3) provides the time-dependent rate of nucleation:

(5) $$v_{t}=K_{N}N_{0}e^{-K_{N}t}$$

The apparent average nucleation rate, v, over the nucleation period $t_{a}$, is defined as:

(6) $$v=\frac{\int_{0}^{t_{a}}v_{t}dt}{t_{a}}=\frac{\int_{0}^{t_{a}}K_{N}N_{0}e^{-K_{N}t}dt}{t_{a}}$$

From Figure 6, it can be indicated that the experimentally determined average reaction rate value is relatively low. Considering Formula (3), this observation implies that the rate constant of nucleation $K_{N}$ is likely also small. Consequently, the exponential term in Formula (5) is approximately equal to 1. Therefore, it can be simplified to the expression for the apparent average nucleation rate:

(7) $$lnv=ln\frac{\int_{0}^{t_{a}}K_{N}N_{0}\times 1dt}{t_{a}}=ln(K_{N}N_{0})=lnK_{N}+lnN_{0}$$

As El-Geassy et al. [13,34] demonstrated, a relatively strong correlation between the apparent average nucleation rate and CO concentration in the gas reactant $C_{CO}$. Therefore, provided $K_{N}$ is considered to be a constant, there should be a functional relationship ($g(C_{CO})$) between $C_{CO}$ and $N_{0}$. Based on Formula (7) and this inferred functional relationship ($g(C_{CO})$) deduced from theoretical and experimental results, it can be simplified rewritten as a constant part (M) and a functional part ($G(C_{CO})$) to further express the correlation on $C_{CO}$:

(8) $$lnv=M+G(C_{CO})$$

**Figure 7:**
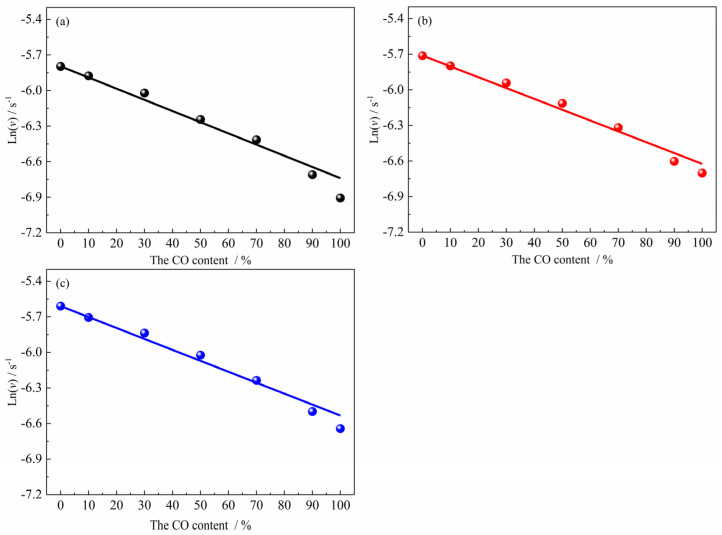
The relationship between average reaction rate and gas composition: (**a**) 1173 K; (**b**) 1273 K; (**c**) 1373 K.

Under the experimental conditions of 1023 K, relatively substantial carbon deposition induced by CO-containing gas reactant during the later stage of the reduction reaction introduces significant systematic deviations between the apparent reaction extent and the reduction extent, resulting in the inability to obtain real-time and accurate reduction extent data. Consequently, the reduction rate at this temperature cannot be analyzed. The experimental results in Figure 6b–d, combined with Formula (8), revealed the corresponding relationship between the apparent average reduction rate of the reaction of ferric oxide samples with H_2_–CO gas mixture and the composition of H_2_–CO gas mixture in the temperature range of 1173 K to 1373 K. It can be found that the logarithm of apparent average reduction rate was basically linear with the CO content, as shown in Figure 7.

(9) $$lnv=lnv_{H_{2}}+N\cdot C_{CO}$$

where N and $v_{H_{2}}$ are the slope of Formula (9) (a constant) and the apparent average reduction rate under pure H_2_ conditions. In addition, since the specific functional relationship ($g(C_{CO})$) between the nucleation rate and the gas reactant composition is not clear. Formula (9) is not derived strictly by a pure mathematical model, but obtained by assuming conditions and combining with experimental results, which can still be applied to predict relatively accurately the apparent average reaction rate under other atmosphere conditions.

**Figure 8:**
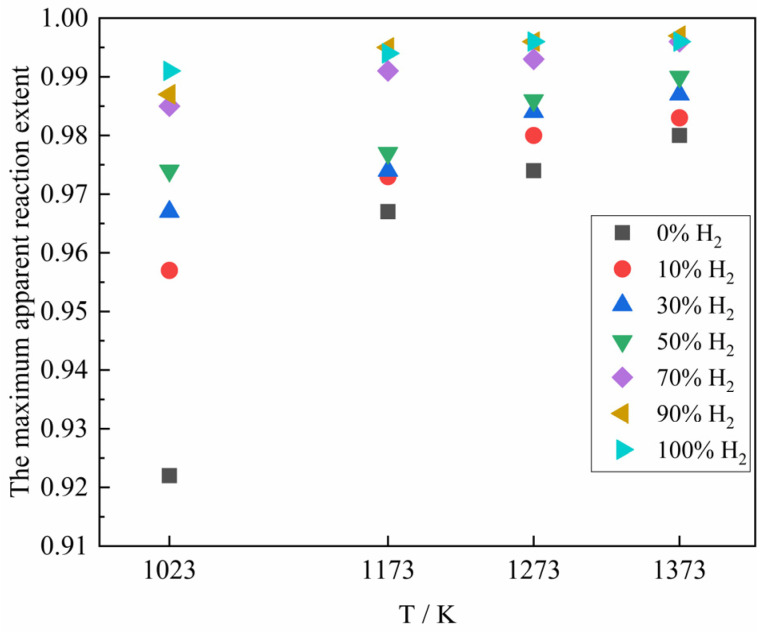
The maximum apparent reaction extent in the reaction process under different conditions.

Figure 8 represents the variation of the maximum apparent reaction extent for the reaction of the ferric oxide sample with the H_2_–CO gas mixture under different conditions. As shown in Figure 8, with the increase of reaction temperature, the maximum apparent reaction extent presented an increasing trend. The possible explanation was that although higher temperatures accelerated the reduction rate and promoted sintering of the formed iron, the resulting structural changes were not sufficient to completely block gas diffusion pathways within the experimental timeframe. Consequently, the gas reactant could still access the reaction interface, allowing the reduction to proceed. The maximum apparent reaction extent of the sample also increased with the increase of H_2_ content in the H_2_–CO gas mixture. Although the increase of H_2_ content would make the sintering situation more serious (the faster kinetics and enhanced diffusion) and affect the gas diffusion, it could still improve the rate and apparent reaction extent of the reduction reaction as a whole. When the gas reactant was pure CO for the reduction reaction, the maximum apparent reaction extent at 1023 K was only 0.922, which may be mainly caused by the following two reasons. Firstly, during the reduction of ferric oxide sample by CO, due to the slow reduction rate of CO, the Fe reduced slowly blocked the pores after a long period of mutual diffusion, and compared with H_2_, the large molecular structure and relatively low diffusion capacity of CO, which eventually made some iron oxides in the center of the sample were not completely reduced to Fe within the reaction time. Secondly, in the presence of Fe, CO as a gas reactant would take place carbon deposition reaction at the same time, resulting in the presence of graphite or cementite in the reduced sample, which was manifested as sample weight gain. In addition, when the H_2_ content in the H_2_–CO gas mixture was higher than 70%, the maximum apparent reaction extent of the sample was basically the same.

In order to further investigate the relationship between the reaction products and the components of the H_2_–CO gas mixture, the carbon deposition phenomenon during the reduction process was briefly analyzed in this paper. As shown in Figure 3, the thermodynamic equilibrium phase diagram of the carbon deposition reaction at different temperatures was presented, and it can be found from the figure that the carbon deposition reaction can only take place under higher CO content at high temperatures. According to previous studies [19], in the presence of Fe, the carbon deposition reaction in H_2_–CO gas mixture may be represented by the following steps, where the generation rate of ${\left(H_{2}\cdot CO\right)}_{ads}^{*}$ is relatively faster than ${\left(CO\cdot CO\right)}_{ads}^{*}$:

(10) $${\left(CO\right)}_{g}+⊡⇄{\left(CO\right)}_{ads}\;or\;{\left(H_{2}\right)}_{g}+⊡⇄{\left(H_{2}\right)}_{ads}$$

(11) $${\left(CO\right)}_{ads}\backslash {\left(H_{2}\right)}_{ads}+{\left(H_{2}\right)}_{g}\backslash {\left(CO\right)}_{g}\to {\left(H_{2}\cdot CO\right)}_{ads}^{*}\;or\;{\left(CO\right)}_{ads}+{\left(CO\right)}_{g}\to {\left(CO\cdot CO\right)}_{ads}^{*}$$

(12) $${\left(H_{2}\cdot CO\right)}_{ads}^{*}\to C+{\left(H_{2}O\right)}_{ads}\;or\;{\left(CO\cdot CO\right)}_{ads}^{*}\to C+{\left(CO_{2}\right)}_{ads}$$

(13) $${\left(H_{2}O\right)}_{ads}\to {\left(H_{2}O\right)}_{g}+⊡\;or\;{\left(CO_{2}\right)}_{ads}\to {\left(CO_{2}\right)}_{g}+⊡$$

where ${\left(H_{2}\cdot CO\right)}_{ads}^{*}$, ${\left(CO\cdot CO\right)}_{ads}^{*}$ and ⊡ are the metastable state of H_2_–CO active molecule, the metastable state of CO–CO active molecule, and the vacant active site on metallic Fe, respectively.

(14) $$3Fe+C\to {Fe}_{3}C$$

**Figure 9:**
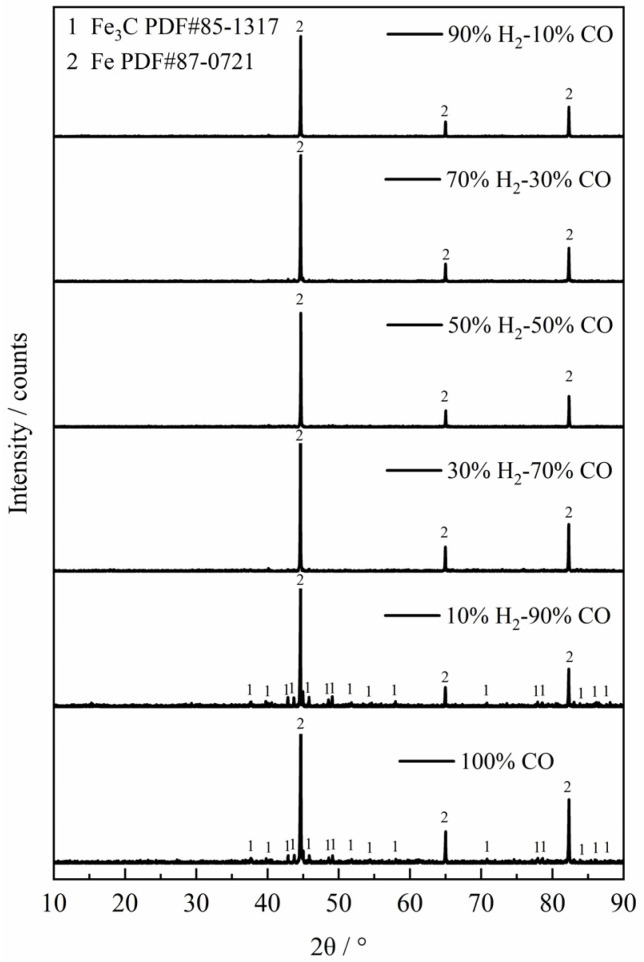
The XRD diffraction patterns of the reaction products at 1023 K.

Overall, it can be found from Figure 5 that the carbon deposition phenomenon was more serious at 1023 K, but with the increase of reaction temperature, the carbon deposition phenomenon decreased rapidly until it almost disappeared. In order to study the carbon deposition phenomenon of the product at 1023 K, the reaction was carried out again under the same experimental conditions as Table 1. When the sample reached the maximum apparent reaction extent, the input of H_2_–CO gas mixture was immediately stopped, and then the Ar gas was introduced, and the thermogravimetric analyzer was quickly cooled, instead of continuing the reaction. The reaction products were analyzed by X-ray diffraction (XRD), and the results are shown in Figure 9. From the figure, it can be found that when the gas reactant with a gas composition of 100% CO and 10% H_2–_90% CO reacted with the ferric oxide sample, there would be a small amount of Fe_3_C in the reaction product. However, as the H_2_ content in the H_2_–CO gas mixture continued to increase, there was basically only metal Fe in the reaction product. Therefore, it can be concluded that when the H_2_ content in the H_2_–CO gas mixture was higher than 30% at 1023 K, the carbon deposition reaction basically does not occur during the reduction reaction, or the C produced by the carbon deposition reaction was rapidly consumed (carbon direct reaction consumption or Boudouard reaction equilibrium shift), but the carbon deposition phenomenon occurred when the H_2_ content was lower than 10%, which is in accordance with Szekely’s results [35].

**Figure 10:**
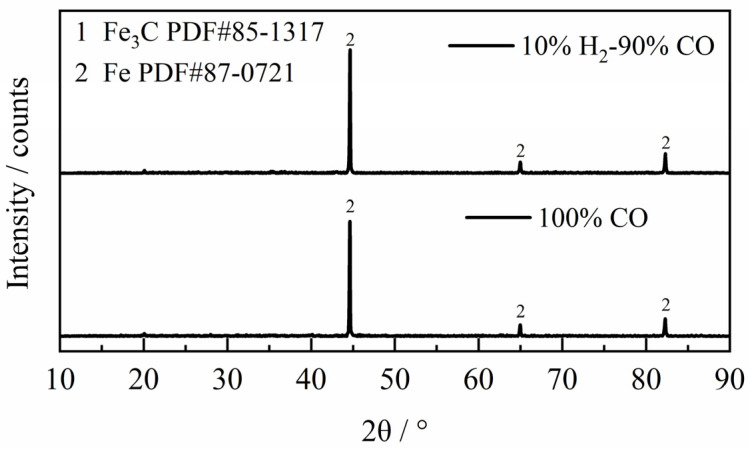
The XRD diffraction patterns of the reaction products at 1173 K.

Further investigations were conducted at 1173 K under an H_2_–CO gas mixture with H_2_ content below 10%. The XRD diffraction pattern of the reaction products (Figure 10) show basically no detectable Fe_3_C phase (PDF#85-1317, the standard X-ray diffraction pattern is shown in Figure S1 of Supporting Information), and the phase of the product was basically metal Fe (PDF#87-0721, the standard X-ray diffraction pattern is shown in Figure S2 of Supporting Information). These results confirm that the reduction stage of the reaction between H_2_–CO gas mixture and ferric oxide sample was basically not accompanied by the occurrence of carbon deposition reaction at 1173 K, consistent with the thermodynamic analysis in Figure 3. In other words, under the same conditions, the carbon deposition phenomenon would be more difficult to occur with the increase of temperature in theory. To summarize, by detecting the phase of the reaction product and combining the apparent reaction extent curves, it can be seen that under the experimental conditions of this study, carbon deposition reaction occurred in the reduction stage (during FeO → Fe) only when the reaction temperature was 1023 K and the content of H_2_ in the H_2_–CO gas mixture was less than 30%, thus affecting the results of experiment.

As mentioned above, the third stage of the reaction is the controlling stage of the whole reaction. Therefore, in order to further explore the reaction behavior of the ferric oxide sample with an H_2_–CO gas mixture, the rate-controlling step of the third stage of the reaction should be clarified, and the apparent activation energy (*E*a) obtained contributes to the understanding of the rate-controlling step. According to the Arrhenius Equation, the value of *E*a can be obtained [36]:

(15) $$k=A_{0}e^{\frac{-E_{a}}{RT}}$$

(16) $$lnk=\ln\left(A_{0}e^{\frac{-E_{a}}{RT}}\right)=lnA_{0}-\frac{E_{a}}{RT}$$

(17) $$\frac{dX}{dt}=A_{0}e^{\frac{-E_{a}}{RT}}\cdot f(X)$$

(18) $$F\left(X\right)=\int_{0}^{X}\frac{dX}{f(X)}$$

where *A*_0_, *k*, *R*, *f*(*X*), *F*(*X*) are the pre-exponential factor, the reaction rate constant, the gas constant, the reaction mechanism model differential form function, and the integral form function, respectively. Separating variables and integrating Formula (17), it can be derived that

(19) $$F\left(X\right)=A_{0}e^{\frac{-E_{a}}{RT}}\cdot t=kt$$

Previous studies [37,38,39,40,41,42,43] provide the proposed mathematical functions for the reaction kinetic model (with a comprehensive summary provided in the previous publication, Reference [19]), which include the majority of probable mechanisms controlling the reaction. These functions will be employed in the mathematical modeling of the reaction kinetic data, with the optimal fitting functions being selected to eventually determine the dominant controlling mechanism of the reaction, where the slope of the fitting function is the rate constant of the reaction (*k*), as shown in Formula (19), and the apparent activation energy is also indirectly obtained by the slope according to Formula (16).

**Figure 11:**
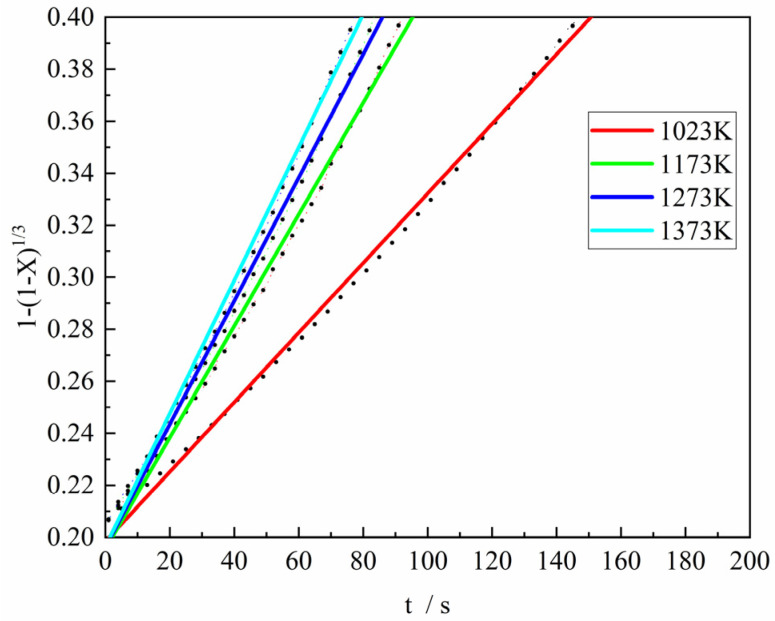
Application of the model of Contracting Volume (R3) at the third stage with 100%H_2._

Figure 11 shows the fitting results of the Contracting Volume model (differential form: $f\left(X\right)=\frac{dX/dt}{k}=3{\left(1-X\right)}^{\frac{1}{3}}$, integral form: $F\left(X\right)=\int_{0}^{X}\frac{dX}{f(X)}=1-{\left(1-X\right)}^{\frac{1}{3}}$) was used to fit the third stage of the reduction reaction between the gas reactant with H_2_ content of 100% and the ferric oxide sample. It can be found from the figure that when the Contracting Volume model was used to fit the experimental data, the consistency was good and the fitting degree was high, and the correlation coefficient (R^2^) of the Contracting Volume model fitting was all greater than 0.99. Consequently, it was considered that the Contracting Volume model was relatively reasonable under this condition. In the same way, the rate constant at each experimental temperature can be derived.

**Figure 12:**
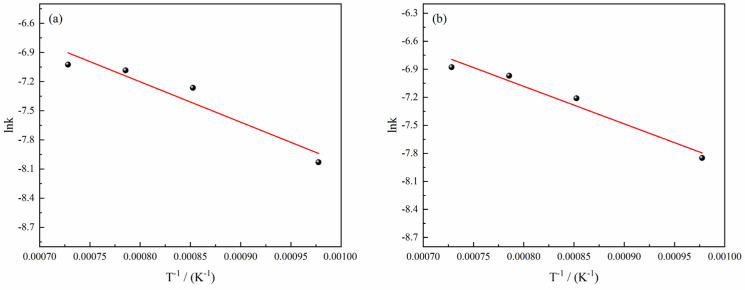
Arrhenius plots of the rate constant for the experiments: (**a**) 100%CO; (**b**) 10%H_2_–90%CO.

**Figure 13:**
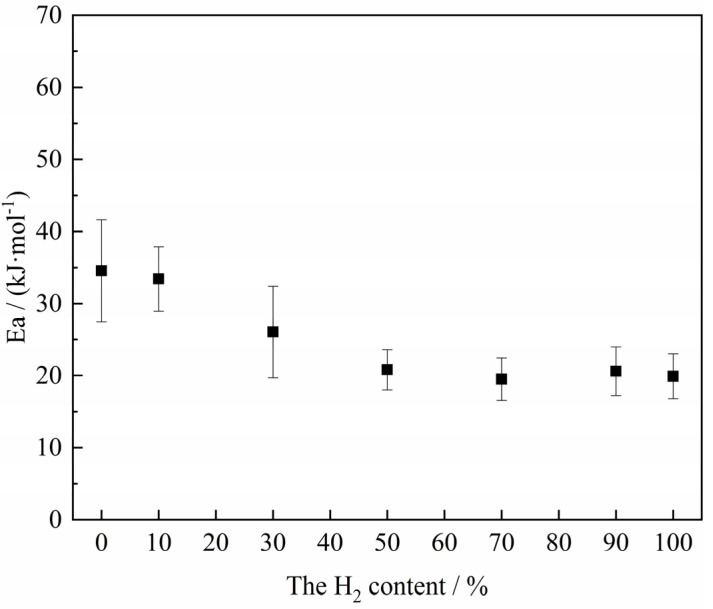
Relation between the composition of the gas reactant and the apparent activation energy.

Figure 12 depicts a linear fitting relationship between the natural logarithm (ln*k*) of the rate constant of the reduction reaction of H_2_–CO gas mixture with ferric oxide sample and the reciprocal of temperature (1/*T*) under different conditions (the complete data diagram is shown in Figure S4 of Supporting Information), from which it can be found that the linear fitting degree between ln*k* and 1/*T* was relatively better, and the slope of the fitting line was the negative value of the quotient of the apparent activation energy and the Molar Gas Constant ($-\frac{E_{a}}{R}$). As a result, the apparent activation energy under different conditions obtained from Figure S4 is shown in Figure 13. The results show that the apparent activation energy exhibited a tendency to decrease with the decrease of H_2_ content in the H_2_–CO gas mixture on the whole. When the H_2_ content in the H_2_–CO gas mixture was less than 10%, the apparent activation energy was maintained at about 34 kJ/mol. According to the empirical relationship between the apparent activation energy and the reaction rate-controlling step studied by Nasr [34] (Gas diffusion: 8–16 kJ/mol; Combined gas diffusion and interfacial chemical reaction: 29–42 kJ/mol; Interfacial chemical reaction: 60~67 kJ/mol; Solid-state diffusion: >90 kJ/mol), the possible rate-controlling step is the combined gas diffusion and interfacial chemical reaction.

When the H_2_ content in the H_2_–CO gas mixture was more than 30%, the apparent activation energy of the reaction was basically maintained at about 20 kJ/mol to 25 kJ/mol, and the possible rate-controlling step was combined gas diffusion and interfacial chemical reaction influenced towards the gas diffusion. The main reason could be that when the H_2_ content in the H_2_–CO gas mixture was high, the sintering and volume shrinkage of the sample was more serious due to its own structural characteristics, resulting in a significant decrease in porosity [12,44], which further made it more difficult for the gas reactant to diffuse to the reaction interface even though H_2_ had a small molecular structure. Moreover, H_2_ had a faster rate of interfacial chemical reaction compared to CO. As a result, the reaction was more inclined to gas diffusion controlling under these atmosphere conditions.

## 4. Conclusions

The reaction behavior of the ferric oxide sample with an H_2_–CO gas mixture was investigated with a thermogravimetric analyzer at 1023 K–1373 K. The experiment results obtained by a series of comprehensive analyses can lead to the following conclusions:

(1) Under conditions of 1023 K with H_2_ content below 30%, the ferric oxide sample reduced by the H_2_–CO gas mixture exhibited a pronounced carbon deposition phenomenon during the reduction stage. Therefore, in order to reduce the influence of carbon deposition, the reaction temperature can be appropriately increased to about 1173 K, or the H_2_ content can be higher than 30%.
(2) The third stage of the reaction was the controlling stage of the whole reaction. Based on the single-step nucleation assumption theory and experimental data, the relationship between the average reaction rate and the gas composition of the H_2_–CO gas mixture was established in the third stage of the reaction, and this relationship can be utilized to guide the optimization of gas reactant composition, thereby achieving an efficient, low-carbon, and sustainable ironmaking process.
(3) Under the present experimental conditions, the apparent activation energy exhibited an overall decreasing trend with increasing H_2_ content in H_2_–CO gas mixture, ranging from 20 to 45 kJ/mol, indicating that the possible rate-controlling step was combined gas diffusion and interfacial chemical reaction with a relatively expressive influence of mass transfer in the gaseous phase. Therefore, even under the condition of relatively high H_2_ content, it is still advisable to enhance the reaction by properly optimizing the gas internal diffusion condition.

## Figures and Tables

**Table 1:** Experimental conditions.

No.	Temperature/K	Flow Rate/(mL/min)		No.	Temperature/K	Flow Rate/(mL/min)	
		H_2_	CO			H_2_	CO
1	1023	0	40	15	1273	20	20
2	1173	0	40	16	1373	20	20
3	1273	0	40	17	1023	28	12
4	1373	0	40	18	1173	28	12
5	1023	4	36	19	1273	28	12
6	1173	4	36	20	1373	28	12
7	1273	4	36	21	1023	36	4
8	1373	4	36	22	1173	36	4
9	1023	12	28	23	1273	36	4
10	1173	12	28	24	1373	36	4
11	1273	12	28	25	1023	40	0
12	1373	12	28	26	1173	40	0
13	1023	20	20	27	1273	40	0
14	1173	20	20	28	1373	40	0

## Data Availability

The original contributions presented in this study are included in the article. Further inquiries can be directed to the corresponding author.

## Supplementary Materials

The following supporting information can be downloaded at: https://www.mdpi.com/article/10.3390/ma18215002/s1, Figure S1: The standard X-ray diffraction pattern of the Fe_3_C (PDF#85-1317); Figure S2: The standard X-ray diffraction pattern of the Fe (PDF#87-0721); Figure S3: The reduction curves for sample with H_2_-CO gas mixture: (a) 1023 K; (b) 1173 K; (c) 1273 K; (d) 1373 K; Figure S4: Arrhenius plots of the rate constant for the experiments: (a) 100%CO; (b) 10%H_2_-90%CO; (c) 30%H_2_-70%CO; (d) 50%H_2_-50%CO; (e) 70%H_2_-30%CO; (f) 90%H_2_-10%CO; (g) 100%H_2_.
