# Supplementary material for: Reaction Behavior of Ultrafine Ferric Oxide Powder with Hydrogen–Carbon Monoxide Gas Mixture
Source: Materials (Basel). 2025 Nov 1;18(21):5002. doi: 10.3390/ma18215002 (PMC12611015; doi:10.3390/ma18215002)
Supplement: Supplementary file 1 [file materials-18-05002-s001.zip › materials-3884132-supplementary.pdf]

Supporting Information

Reaction Behavior of Ultrafine Ferric Oxide Powder with Hy-drogen–Carbon Monoxide Gas Mixture

Xudong Mao [1, 2, 3]

[1] School of Resources & Environment, Nanchang University, Nanchang 330031, China; maoxudong_ustb@163.com
[2] Jiangxi General Institute of Testing and Certification, Nanchang 330052, China
[3] State Key Laboratory of Advanced Metallurgy, University of Science and Technology Beijing, Beijing 100083, China

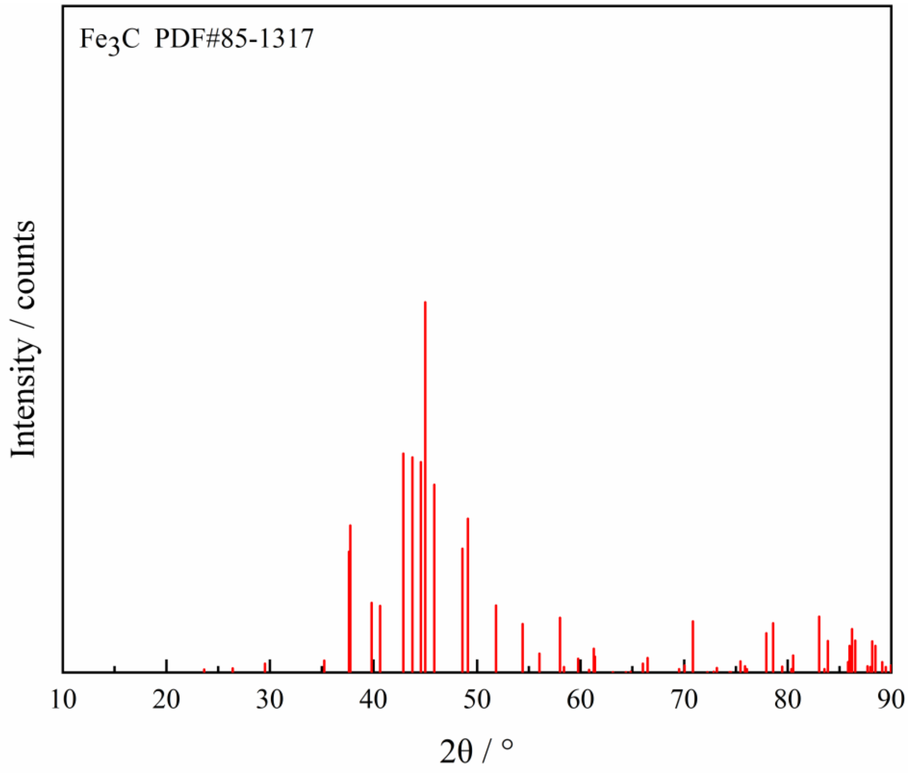

Figure S1 The standard X-ray diffraction pattern of the Fe_3C (PDF#85-1317).

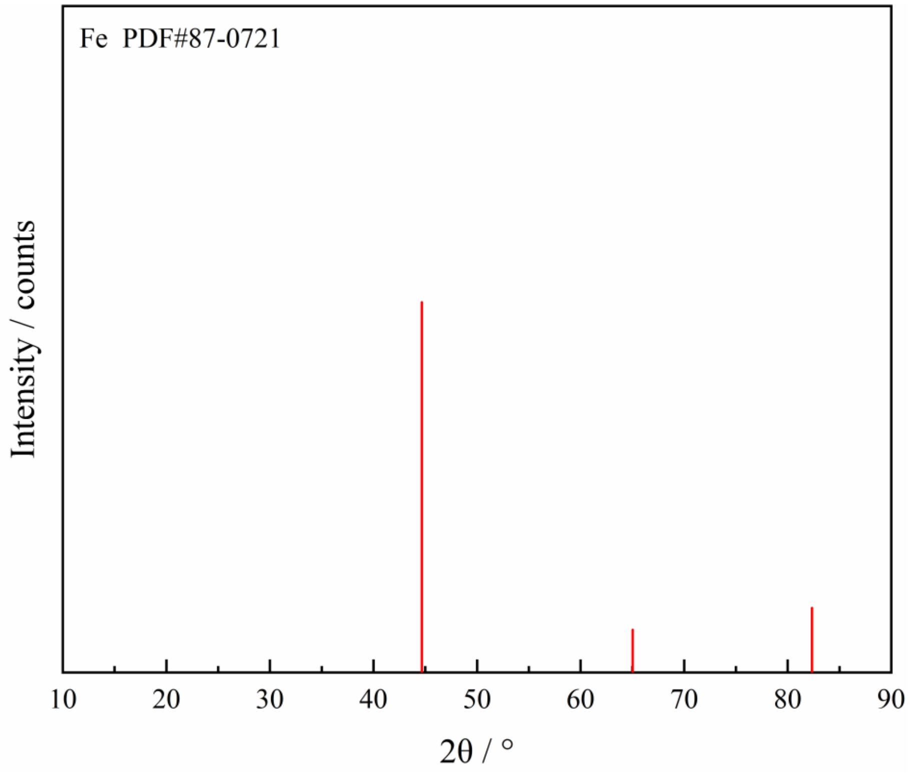

Figure S2 The standard X-ray diffraction pattern of the Fe (PDF#87-0721).

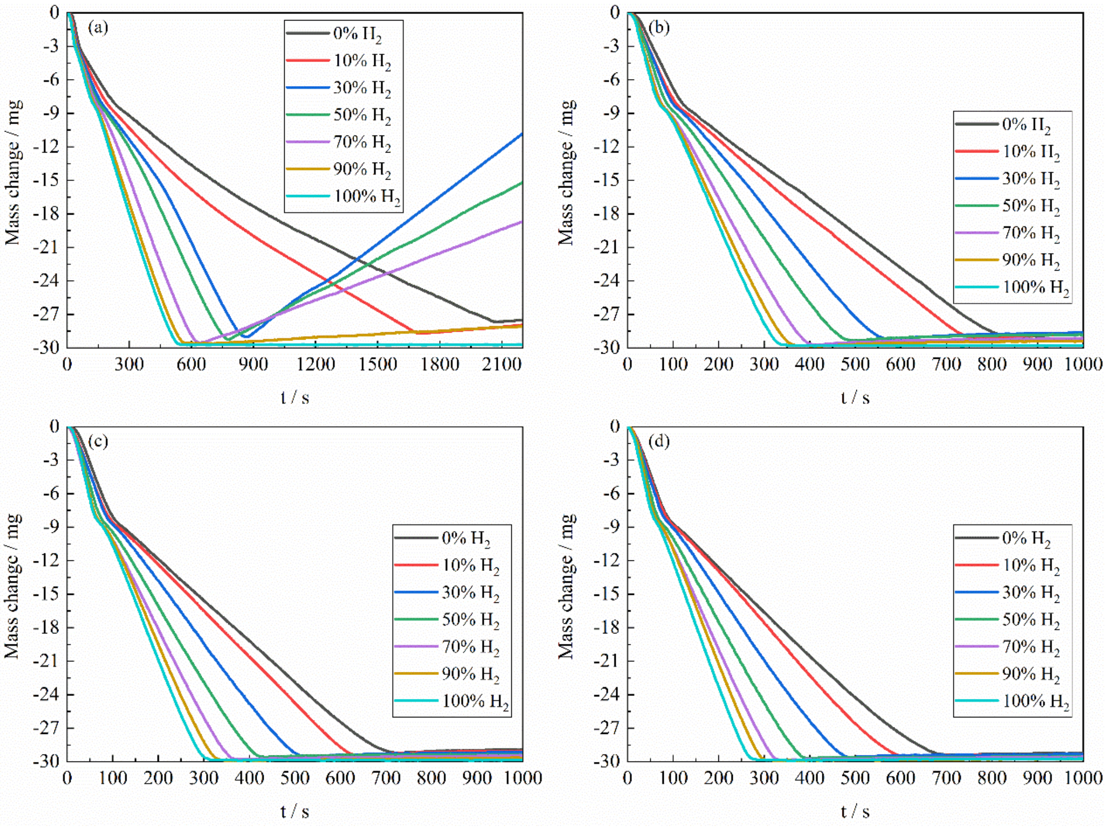

Figure S3 The reduction curves for sample with H_2-CO gas mixture: (a) 1023

K; (b) 1173 K; (c) 1273 K; (d) 1373 K.

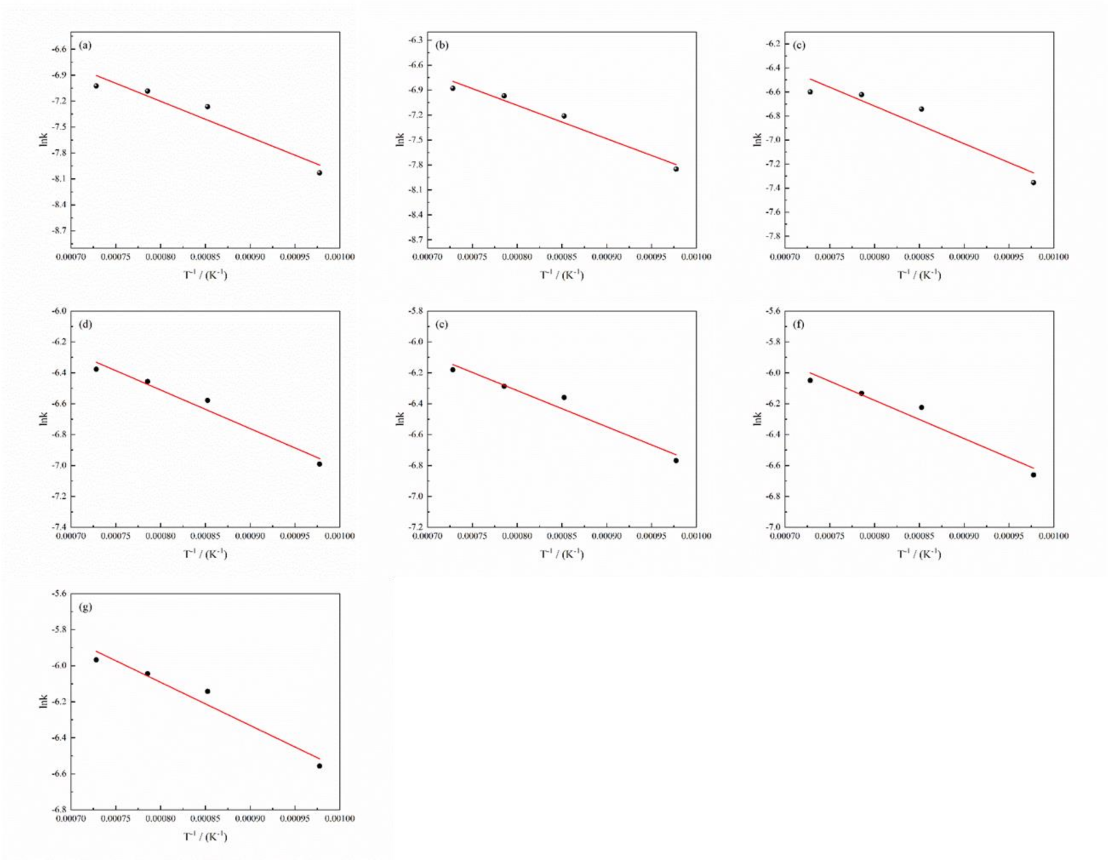

Figure S4 Arrhenius plots of the rate constant for the experiments: (a) 100%CO; (b) 10%H_2-90%CO; (c) 30%H_2-70%CO; (d) 50%H_2-50%CO; (e) 70%H_2-30%CO; (f) 90%H_2-10%CO; (g) 100%H_2
